# Encapsulation of Copper Nanoparticles in Electrospun Nanofibers for Sustainable Removal of Pesticides

**DOI:** 10.1021/acsami.3c00849

**Published:** 2023-04-16

**Authors:** Ana Isabel Quilez-Molina, Suset Barroso-Solares, Violeta Hurtado-García, José Alejandro Heredia-Guerrero, María Luz Rodriguez-Mendez, Miguel Ángel Rodríguez-Pérez, Javier Pinto

**Affiliations:** † Cellular Materials Laboratory (CellMat), Condensed Matter Physics, Crystallography, and Mineralogy Department, Faculty of Science, University of Valladolid, Campus Miguel Delibes, Paseo de Belén n° 7, Valladolid 47011, Spain; ‡ BioEcoUVA Research Institute on Bioeconomy, Calle Dr. Mergelina, Valladolid 47011, Spain; § Archaeological and Historical Materials (AHMAT) Research Group, Condensed Matter Physics, Crystallography, and Mineralogy Department, Faculty of Science, University of Valladolid, Campus Miguel Delibes, Paseo de Belén n° 7, Valladolid 47011, Spain; ∥ Instituto de Hortofruticultura Subtropical y Mediterránea “La Mayora”, Universidad de Málaga-Consejo Superior de Investigaciones Científicas (IHSM, UMA-CSIC), Bulevar Louis Pasteur 49, Málaga 29010, Spain; ⊥ Group UVaSens, Escuela de Ingenierías Industriales, Universidad de Valladolid, Paseo del Cauce, 59, Valladolid 47011, Spain

**Keywords:** PCL, chlorpyrifos, copper oxide, Cu_2_O, catalysis, electrospinning, reusability

## Abstract

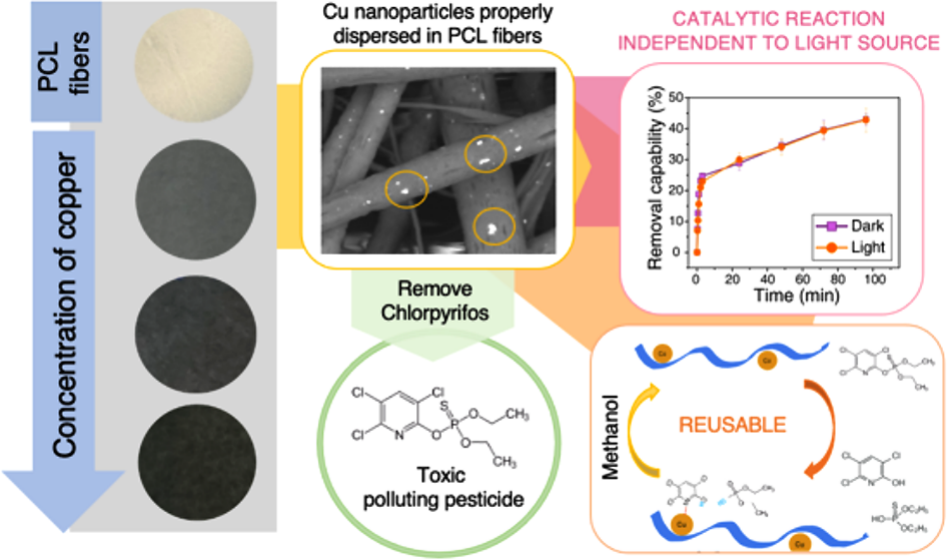

The excellent catalytic properties of copper nanoparticles (CuNPs) for the degradation of the highly toxic and recalcitrant chlorpyrifos pesticide are widely known. However, CuNPs generally present low stability caused by their high sensitivity to oxidation, which leads to a change of the catalytic response over time. In the current work, the immobilization of CuNPs into a polycaprolactone (PCL) matrix via electrospinning was demonstrated to be a very effective method to retard air and solvent oxidation and to ensure constant catalytic activity in the long term. CuNPs were successfully anchored into PCL electrospun fibers in the form of Cu_2_O at different concentrations (from 1.25 wt % to 5 wt % with respect to the PCL), with no signs of loss by leaching out. The PCL mats loaded with 2.5 wt % Cu (PCL-2.5Cu) almost halved the initial concentration of pesticide (40 mg/L) after 96 h. This process was performed in two unprompted and continuous steps that consisted of adsorption, followed by degradation. Interestingly, the degradation process was independent of the light conditions (i.e., not photocatalytic), expanding the application environments (e.g., groundwaters). Moreover, the PCL-2.5Cu composite presents high reusability, retaining the high elimination capability for at least five cycles and eliminating a total of 100 mg/L of chlorpyrifos, without exhibiting any sign of morphological damages.

Pesticides are a necessary tool for reducing the damage to crops from pests and for ensuring food production. However, most pesticides are toxic and highly recalcitrant in the environment with a negative impact on human health and ecosystems.^1,2^ Indeed, pesticide residues have been found in fruits and vegetables, as well as in drinking water, which supposes a potential risk for the global population today.^1,2^ The massive and uncontrolled accumulation of pesticides in developing countries and countries with economies in transition have complicated the management of these highly hazardous substances.^3,4^ This critical situation has fostered the implementation of new strong policies and legislation with the aim to eliminate obsolete pesticides and apply rapid environmental assessment tools in highly contaminated sites.^1,5,6^ Among pesticides, organophosphorus compounds form the largest group of chemical pesticides, accounting for approximately 34% worldwide, and have been widely used for more than 50 years by farmers for protecting crops.^7^ Their attractiveness and large usage are due to their low price, effectiveness, and broad activity.^8^ Chlorpyrifos (O,O-diethyl O-(3,5,6-trichloro-2-pyridinyl) phosphorotionate) is the most popular organophosphate pesticide because of its broad-spectrum activity (e.g., insecticide, acaricide, and miticide properties^8,9^). However, chlorpyrifos is extremely toxic for a wide range of nontarget organisms, especially aquatic.^9,10^ Poisoning from chlorpyrifos provokes serious health problems in nervous, respiratory, and cardiovascular systems even at low exposure (maximum of admissible quantity between 0.0001 and 0.0005 mg/L).^9,11^ Its detection in the environment, in foods, and in all kinds of agricultural products has created a strong awareness of the health risks that the use of this product entails.^9,12^ In addition, the high stability and accumulation characteristics affect a huge area that covers 24 km away from the place of application.^9,11,13^ Recently, The European Food Safety Authority (EFSA) approved the banning of chlorpyrifos in January 2020.^9^ However, this pesticide is still in use in some countries like India, China, and Japan.^9^ In addition, despite the ban, chlorpyrifos biomarkers are still detected in large amounts in countries such as France and Spain.^10,14^

There are many methods to eliminate organophosphates in wastewater, including biological treatments (e.g., fungal activity^15^), direct UV light,^4^ oxidation processes,^16^ enzymatic activity,^15,17^ and adsorption.^18,19^ Indeed, new research lines focused on the development of new active materials with the capability of absorbing or degrading these hazardous compounds have emerged in the past few years. Nanomaterials are the most promising candidates for this scope due to their high surface area and chelating capability.^1,20^ The catalytic properties of metallic nanoparticles have been widely studied for several applications. The literature shows different metallic nanoparticles with the capability to degrade chlorpyrifos, such as silver, copper, iron, and gold.^21−23^ In many cases, the degradation effectivity of the pesticide has been enhanced by combining these nanoparticles with oxidizing agents and with light irradiation in the UV or visible ranges.^4,11,13^ However, even though the disadvantages of free nanoparticles are well-known (e.g., leaching out and agglomeration), few articles consider the immobilization of these nanoparticles in a matrix.^23,24^ Moreover, the necessity of a light source to undergo this catalytic process can limit the applicability of the material only to superficial waters, precluding aquifers and groundwaters.^25^

In general, copper nanoparticles (CuNPs) and Cu-based compounds have been widely used for water remediation applications, including wastewater^26^ and antibiotics pollution.^27^ The chemical features of copper provide a high affinity toward the thionate or oxonate groups present in organophophonates interacting via covalent bond, highly useful for the development of emerging pesticide detectors.^28,29^ Besides, the use of copper species for chlorpyrifos degradation/removal is widely reported elsewhere.^17,21,22^ In most of these systems, copper is commonly present in the oxidation state of +2 due to its high susceptibility to undergoing air and solvent oxidation.^17,30^ Literature reports show that the oxidation from Cu (0) to Cu_2_O (oxidation state +1) naturally occurs after a few minutes of air exposure, followed by a further oxidation to CuO (oxidation state +2).^31,32^ The progressive metal oxidation is an undesirable effect that hampers constant long-term operation.

A novel strategy to retard the oxidation and to obtain a sustained response over time is the use of polymers as capping agents.^33−35^ In the cases found in the literature, the polymer matrices include multiple reagents or several fabrication steps.^31,36,37^ On the contrary, electrospinning is a modern technique that allows the fabrication of highly active metal/polymer composite mats in a single step.^31,38,39^ Over the years, this technique has gained popularity due to its simple and versatile process, which consists of the production of fibers by applying a high-voltage electric field to a polymer solution injected from a syringe.^39,40^ In the literature, these fibers have encapsulated active molecules such as antioxidants and metallic nanoparticles, providing tailoring properties, which can be applied in multiple fields, like food packaging, wound dressing, drug delivery, etc.^40,41^ The high potential of electrospun fibers for pollutant removal application is linked to the large specific surface area, which is highly convenient to enhance the interaction with the target compound.^38,42^ Among other polymers, polycaprolactone (PCL) presents excellent electrospinnable properties, being also a biodegradable, biocompatible, and water-resistant polymer which is extensively used for long-term drug-delivery systems.^2,43^ For instance, Chen et al.^35^ obtained polymeric membranes with excellent antibacterial properties by combining PCL composite with copper oxide nanoparticles using electrospinning. In the field of water treatment, electrospun fibers of PCL have been proven to remove heavy metals and to separate oil stable emulsions when inorganic compounds such as kaolin clay^44^ or silica nanoparticles,^45^ respectively, are embedded in the matrix.

In this work, nanocomposite PCL electrospun fibers incorporating copper are presented for the first time as a new, promising method to degrade chlorpyrifos in an aqueous environment. PCL microfibers act as capping agents, preserving copper largely from oxidation reactions. Interestingly, the reaction mechanism of the chlorpyrifos degradation was not affected by the light source, obtaining similar results in dark and light conditions, which indicates that this material could be applied in different environments. Last but not least, the obtained composite fibers were shown to be reusable for at least five cycles without exhibiting any degradation effect and fully retaining their capability to degrade chlorpyrifos.

## Results and Discussion

### Morphologic Analysis of the Fibers

**Figure 1:**
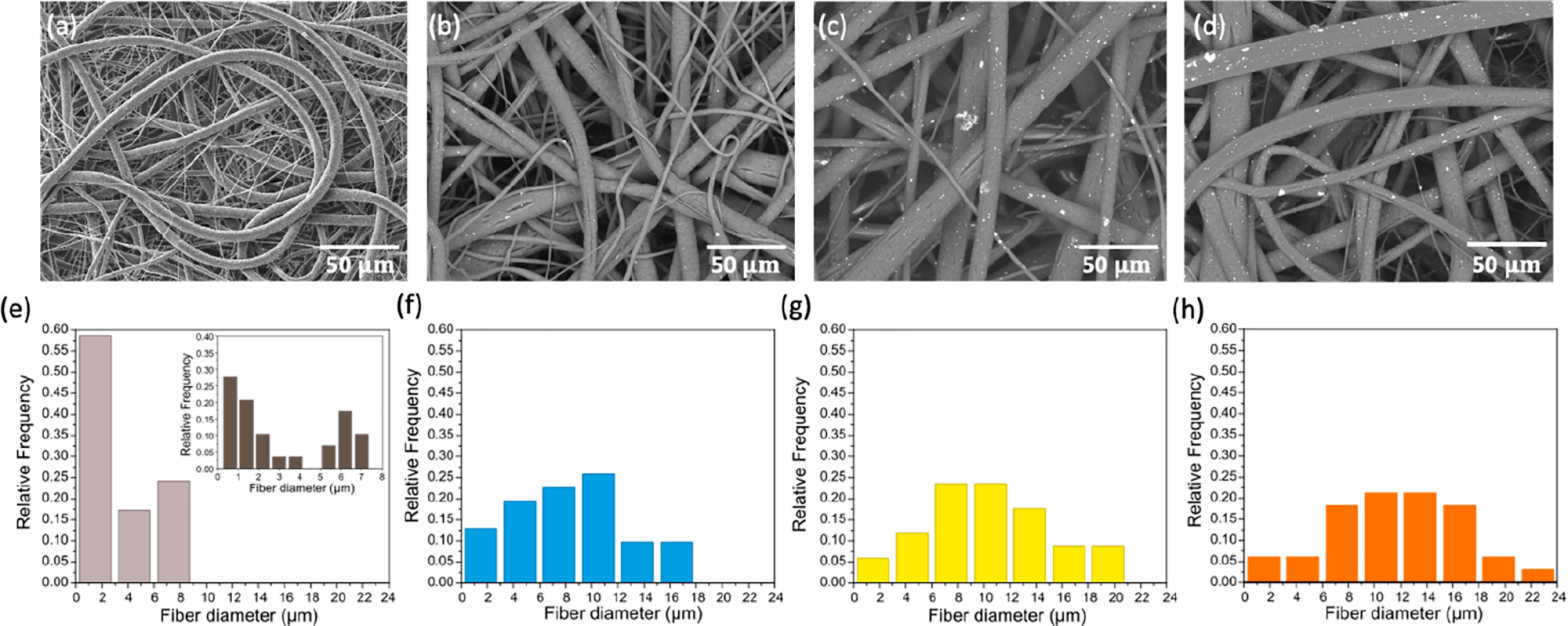
SEM images of (a) PCL (b) PCL-1.25Cu, (c) PCL-2.5Cu, and (d) PCL-5Cu. The histograms of fiber size distribution of (e) PCL, (f) PCL-1.25Cu, (g) PCL-2.5Cu, and (h) PCL-5Cu.

SEM micrographs of the neat PCL mat and PCL mat samples containing 1.25, 2.50, and 5.00 wt % Cu nanoparticles are displayed in Figure 1a–d. In all cases, electrospun fibers were bead-free and randomly distributed in the matrix. Higher magnification micrographs of the mats (Figure S1) showed that the fibers exhibited some cracks as a result of the solvent evaporation at high relative humidity (∼33%, according to results reported elsewhere.^46^ In Figure 1e and inset, neat PCL mats clearly exhibited a bimodal distribution of fiber diameter, ranging from approximately 0.2–4 and 5–7.5 μm. The histograms of the copper-containing samples reported in Figures 1f–h displayed a Gaussian distribution with an increment of the diameter size with the content of copper. This increase in diameter size reached maximum values of ∼23 μm for PCL-5Cu, against <10 μm of fiber size observed in neat PCL. As reported previously,^41,47^ this trend results from an increase in the viscosity of the spun due to the presence of nanoparticles. The maximum concentration of copper selected was 5 wt % because the high viscosity reached above this concentration hindered the production of the electrospun fibers. The SEM images of PCL-1.25Cu, PCL-2.5Cu, and PCL-5Cu showed white spots ubiquitously distributed in the electrospun fibers that increased with the concentration of copper.

**Figure 2:**
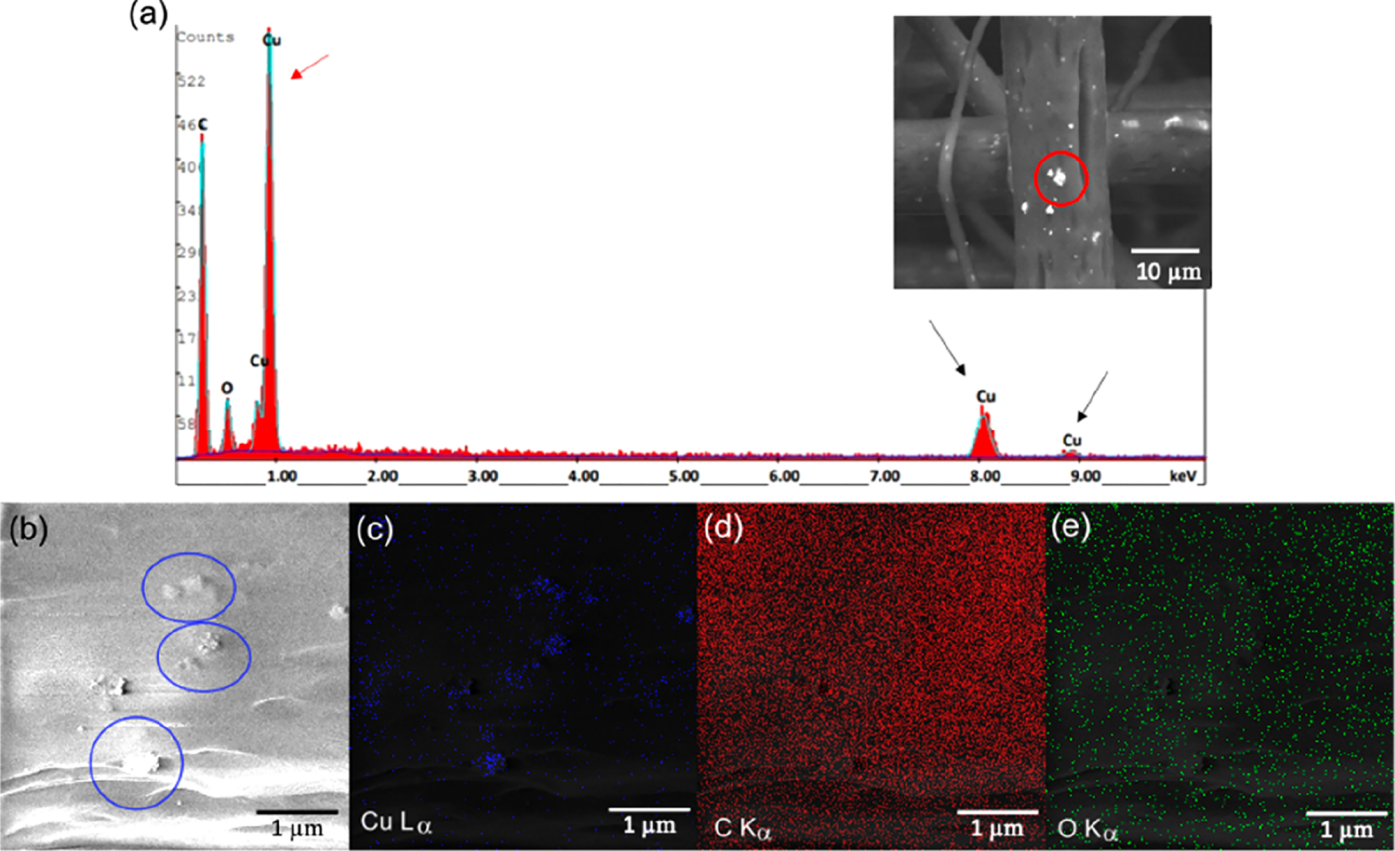
(a) EDS spectrum showing X-ray lines of Cu (*L*_*α*_ = 0.93 keV, *K*_*α*_ = 8,04 keV, and *K*_*β*_ = 8.91 keV), respectively, indicated with red and black arrows, carbon (*K*_*α*_ = 0.27 keV), and oxygen (*K*_*α*_ = 0.52 keV). The inset shows the SEM image of the PCL fiber containing CuNPs under study (b) SEM image of the surface of a PCL-2.5Cu electrospun microfiber with some agglomerates of Cu nanoparticles indicated with blue circles; and the corresponding EDS maps for (c) Cu (*L*_*α*_ = 0.93 keV), (d) carbon (*K*_*α*_ = 0.27 keV), and (e) oxygen (*K*_*α*_ = 0.52 keV), mapping as blue, red, and green spots, respectively.

Figure 2a exhibits the energy-dispersive X-ray spectroscopy (EDX) spectra of the elements present in the spot indicated with a red circle in the inset SEM image of the PCL-2.5Cu fiber. Herein, the typical EDX profile of pure copper appeared, composed of an intense peak (*L*_*α*_ = 0.93 keV), and weaker signals from the K shell (*K*_*α*_ = 8.04 keV and *K*_*β*_ = 8.91 keV). The typical X-ray emission peaks of carbon (*K*_*α*_ = 0.27 keV) and oxygen (*K*_*α*_ = 0.52 keV) belonging to the PCL matrix were also visible. The elemental Cu mapping of the PCL-2.5Cu mats showed that copper nanoparticles were easily detectable in electrospun microfibers (Figures 2b, c). The SEM-EDX images confirmed that CuNPs were well-distributed in overall fiber surface or in the form of aggregates in some determined points. The EDX composition maps represented in Figure 2d, e evidenced that the matrix was compounded mainly by carbon (*K*_*α*_ = 0.27 keV) and oxygen (*K*_*α*_ = 0.52 keV), as expected.

### Chemical Analysis

**Figure 3:**
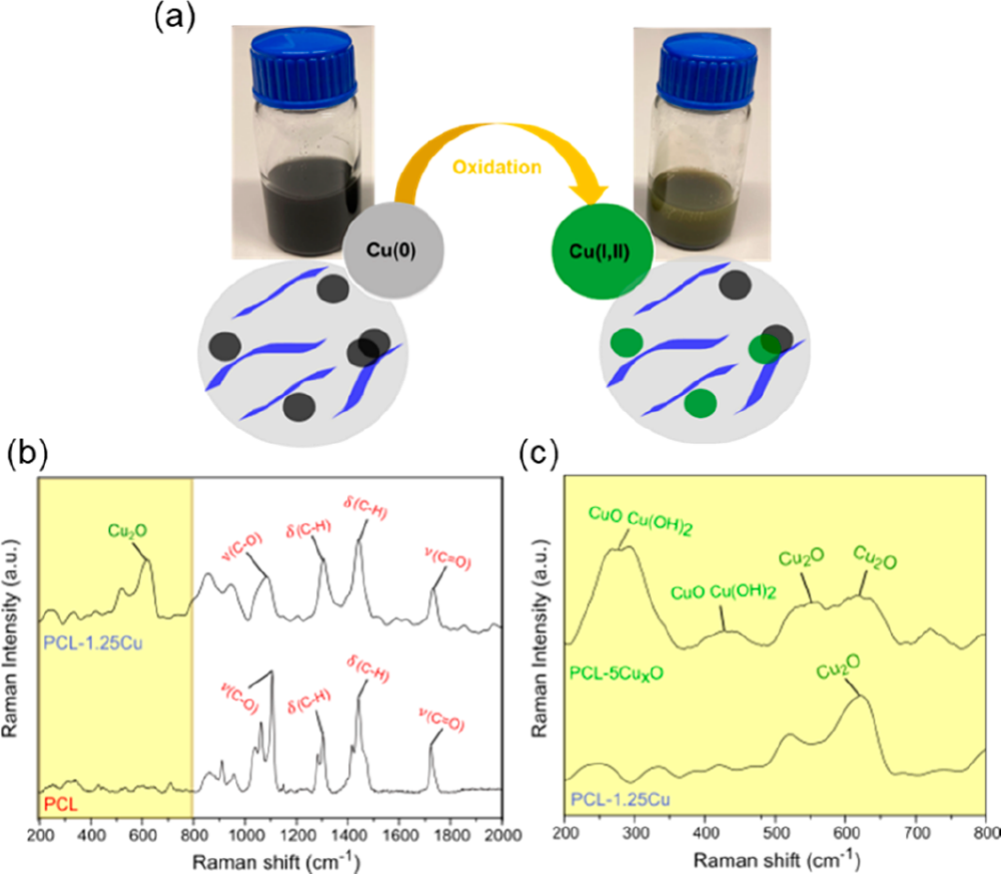
(a) Schematic representation of the color changes observed due to the oxidation reaction of the copper nanoparticles. (b) Raman spectra of the neat PCL and PCL-1.25Cu ranging from 200 to 2000 cm^–1^. (c) Raman spectra of the PCL-1.25Cu and PCL-5Cu_*x*_O samples in the range of 200–800 cm^–1^.

The chemical characterization of the PCL and PCL-Cu samples was first attempted with FTIR spectroscopy and X-ray diffraction (XRD) without providing clear insights about the copper nanoparticles, as reported in Supporting Information (Figure S2).^31,48^ In addition to this, the presence of oxidized species of copper in samples was evaluated through Raman spectroscopy. It is well-known that the use of zerovalent copper nanoparticles is highly limited by the inherent tendency to oxidize to Cu(I) and Cu(II) oxides under ambient reaction conditions.^49^ Indeed, during the storage, the PCL-Cu solutions became greenish over time as a result of the oxidation of copper,^31^ see the scheme in Figure 3a. Electrospun fibers fabricated with the oxidized solution of PCL-5Cu, labeled PCL-5Cu_*x*_O, were used to identify the peaks related to the copper oxides and to determine the oxidation state of the samples.

The Raman spectra of PCL, PCL-1.25Cu, and PCL-5Cu_*x*_O are evaluated in Figure 3b, c. This spectroscopic technique is highly reliable for determining the purity of the copper in the mats because copper oxides exhibit Raman active vibrational modes.^31,49^ In Figure 3b, the Raman spectra of PCL display the characteristic peaks of the carbonyl group and methylene group located at 2910 and 1720 cm^–1^, respectively. The bands associated with the bending vibration of CH arise at 1443 cm^–1^, and 1300 cm^–1^, while the stretching vibration band of COC appears at 1110 cm^–1^.^48^ In the same Figure, the Raman spectra of PCL-1.25Cu present the typical peaks of the PCL polymer except for the strong band in the range from 300 to 670 cm^–1^ (Figure 3c). According to the literature, this band is claimed to correspond to the copper(I) oxide Cu_2_O.^31^ On the contrary, three clear Raman peaks located at 615 cm^–1^, 540 cm^–1^, and 295 cm^–1^ can be easily observed in the spectra of PCL-5Cu_*x*_O (Figure 3c). These peaks correspond to the oxidizing species Cu_2_O and CuO, with oxidation states +1 and +2, respectively.^31,49^ The lack of these peaks in the spectra of the PCL-1.25Cu mat demonstrated that the polymer prevents copper oxidation to a great extent. The capability of polymers to provide long-term stability to copper was also proven in Morioka et al.^33^ with polyvinylpyrrolidone (PVP). Moreover, it was proven that the initial CuNPs employed in this work present no oxidation (see the Supporting Information, Figure S3). Also, no changes on the Raman spectra of the electrospun fibers were found after several weeks stored. Accordingly, it has been proven that the oxidation of the Cu NPs occurs in the solutions prepared for the fabrication of the fibers, as a consequence of the high solubility of atmospheric oxygen in chloroform, which oxidized the Cu (0) nanoparticles.^33,50^ This makes necessary to produce the fibers soon after the solutions preparation. A further chemical analysis was performed by inspecting the XPS spectra of PCL-1.25Cu and PCL-5Cu_*x*_O samples after a couple of months from the fabrication; see Figure S4 of the Supporting Information. Herein, the Auger LMM signals confirmed the presence of Cu (I) in PCL-1.25Cu, while Cu (II) was the only species observed in PCL-5Cu_*x*_O. This indicated that the polymer matrix efficiently preserved CuNPs from oxidation.

### Study of the Pesticide Removal and Reaction Mechanism

**Figure 4:**
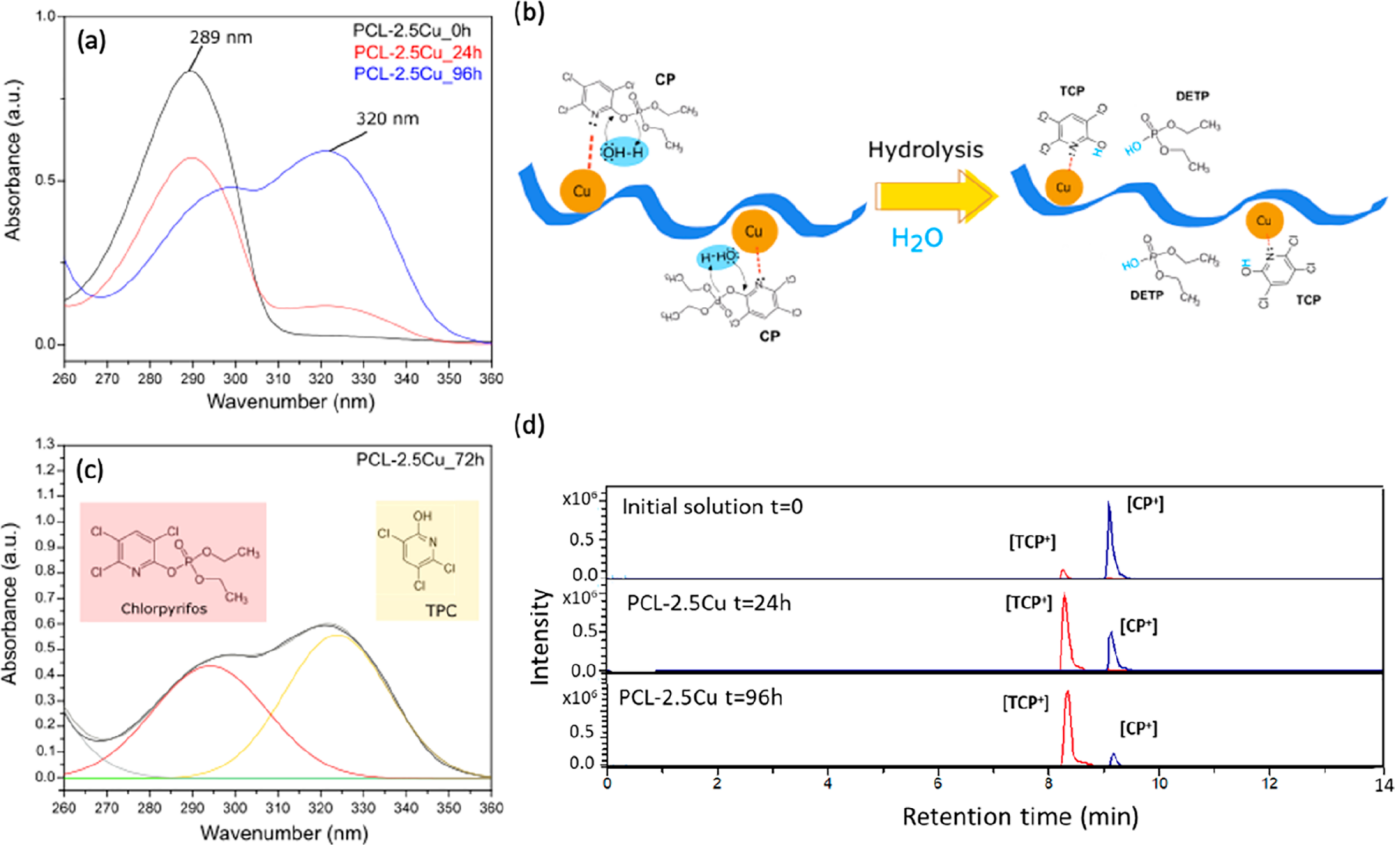
(a) UV–vis spectra of the Chlorpyrifos solution after being in contact with the PCL-2.5Cu sample at different reaction times of 0, 24, and 96 h, ranging from 260 to 360 nm. (b) Scheme of the reaction between the copper nanoparticles embedded in the PCL microfibers, represented as the blue string, and the pesticide. (c) Deconvoluted peaks of the UV–vis band corresponding to CP (in red) and TPC (yellow) of the Chlorpyrifos solution after being in contact with PCL-2.5Cu for 72 h. (d) Chromatogram of the initial solution (*t* = 0), the solution measured after PCL-2.5Cu being immersed for 24 and 96 h. The red peak corresponds to the subproduct TPC^+^, while the blue peak belonged to chlorpyrifos (CP^+^)

The high interest in applying copper as a catalyst for wastewater treatment is linked to its superior catalytic activity and reactivity in degrading organic pollutants.^11,21^ For the study of the catalytic mechanism of PCL-Cu mats, 5 mg of PCL mats (1 × 1.2 cm^2^ with an average thickness of ∼0.3 mm) was put to shake with the reaction solution of 40 mg/L CP in water:ethanol:methanol (2:1:1). This concentration was selected to simulate the maximum concentration of pesticide found in natural waters, 37.4 mg/L. More specifically, in the pond water, paddy field water, tube-well water, and river water at Nagarpur and Saturla Upazila cities (Bangladesh).^10^ The changes in concentration of the pesticide over time were evaluated by monitoring the intensity of the characteristic UV–vis absorbance peak at 289 nm using a UV–vis spectrophotometer.^22,51^Figure 4a displays the UV spectra of the solution before the sample PCL-2.5Cu was immersed and after 24 and 96 h. The spectra indicated that the characteristic peak of chlorpyrifos (289 nm) decreased gradually over time, while a new peak at ∼320 nm appeared after 24 h. The UV–vis spectra of the CP solution after the immersion of the other PCL-Cu fibers, PCL-1.25Cu, and PCL-5Cu, exhibited the same behaviors; see Figure S5a, b. The formation of a new product (peak 320 nm) strongly suggested that a catalytic reaction between the PCL-Cu mats and pesticide occurred.

For comparison, the same procedure was performed with 5 mg of Cu powder introduced in paper wettable pouches, showing the same UV-spectra in Figure S5c. This indicated that the Cu participated in the catalytic degradation reaction, while PCL acted as an inert matrix. The incorporation of nanoparticles into electrospun fibers is a widely applied strategy to avoid the strong tendency of free metallic nanoparticles to aggregate and to provide a high contact surface area to the catalyst.^52,53^ Therefore, a proper dispersion of copper nanoparticles into PCL electrospun fibers could ensure a high performance of the catalytic response.^41,53^ Moreover, the oxidation state of the copper nanoparticles embedded in the fibers did not compromise the catalytic response. These hybrid systems facilitate the transport and recovery of the metallic catalyst, which is a highly difficult task with free nanoparticles.^41^ This effect will be thoroughly discussed in the next sections.

To the best of our knowledge, the catalytic mechanism of chlorpyrifos degradation using Cu_2_O or Cu(I) has not yet been reported yet. However, observing the high similarities in the UV–vis spectra, it can be assumed that the catalytic mechanism is similar to those reported with Cu (II).^11,21^ According to this, in the pesticide degradation, the metal was coordinated with the pyridinyl N atoms of CP to promote the hydrolysis in the presence of water molecules and cleavage of the P–O bond, resulting in the degradation products 3,5,6-trichloropyridinol (TPC) and diethyl thiophosphate (DETP)^17,22^ (see the scheme of Figure 4b). The TPC product presents a characteristic UV–vis peak at 320 nm, while DETP was not visible through this technique. The characteristic UV–vis band of TPC was assigned to the deconvoluted peaks, represented in Figure 4c. TPC is demonstrated to be less toxic and not mutagenic with respect to CP.^22^ These results were confirmed using chromatography–mass spectroscopy in the ESI positive mode (UHPCL-MS). The chromatograms are represented in Figure 4d, while the mass spectrum that contains the fragmentation patterns of the organic molecules is reported in Figure S6 (see the Supporting Information). In the chromatograms, the characteristic peak of chlorpyrifos (CP^+^) appears in blue with a retention time equal to 9.1 min, while the peak belonging to the byproduct (TPC^+^) is located at 8.3 min of retention time in red. As expected, the intensity of the CP peak decreased with time, while TPC increased, and no other peaks corresponding to other subproducts appeared.

The chromatogram of the initial chlorpyrifos (CP) solution evidenced the presence of the subproduct TPC in low quantity (Figure 4d). This reveals that the pesticide undergoes minor hydrolysis during storage.^11^ In concordance with this, in Figure S7a, the UV–vis spectra of the CP solution after 96 h manifested a slight increase of the peak at 320 nm and a reduction of the main peak of chlorpyrifos. Overall, the pesticide concentration decreased by less than 1 mg/L, indicating that the loss of CP was insignificant. Interestingly, when the neat PCL mat was immersed in the pesticide solution, the characteristic peak of the chlorpyrifos decreased; see Figure S7b. During this process, the peak belonging to the degradation products of chlorpyrifos located at 320 cm^–1^ did not arise, which confirmed that the loss of the pesticide was due to an adsorption process. In the study of Hinestroza et al.,^54^ PCL fibers were shown to remove other water pollutants through hydrogen bond interaction. The lack of hydrogen donor groups among reagents could indicate that the interaction could be through van der Waals forces.

### Removal Efficiency of the Pesticide

**Figure 5:**
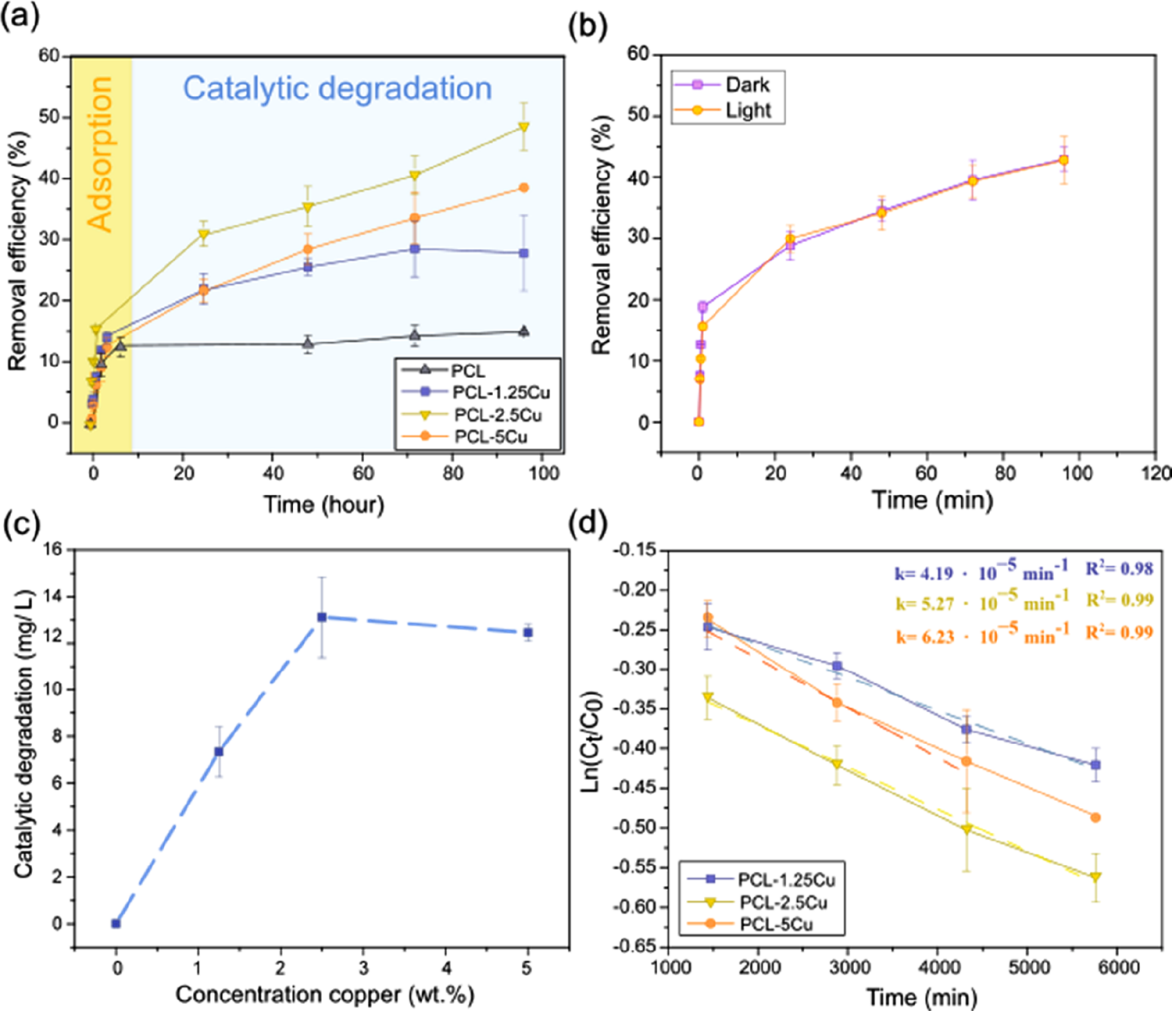
(a) Removal efficiency of PCL, PCL-1.25Cu, PCL-2.5Cu, and PCL-5Cu for 96 h. The yellow shaded area indicates the adsorption stage, whereas the catalytic degradation is highlighted in light blue. (b) Removal efficiency under light and dark conditions of PCL-2.5Cu mats. (c) Catalytic degradation of CP (mg/L) with the concentration of copper in the matrix. (d) Kinetic curves of the catalytic reaction and the first constant rates.

The capability of the produced samples to remove the pesticide was evaluated by immersing the PCL mats in a CP solution of 40 mg/L. The removal efficiency (%) versus time was plotted for the PCL and PCL-Cu mats in Figure 5a. The results show that all samples have removed 15% of CP after the first 5 h. For PCL, this value remained constant for the next 96 h, while the response of PCL-1.25Cu, PCL-2.5Cu, and PCL-5Cu samples continued to rise to a maximum of 28.5%, 48.3%, and 35.0%, respectively. The removal curves in the first experimental hours (<24 h), identical in all samples, corresponded to the adsorption of pesticide in the polymer matrix. The maximum adsorption capacity (*q*_max_) of PCL corresponded to 6.38 ± 0.09 mg chlorpyrifos/g sample, which means the removal of 15% of chlorpyrifos at the experimental conditions. The samples outperformed the *q*_max_ (0.81 mg CP/g sample) obtained in other electrospun fibers, such as cellulose fiber filled with 10 wt % graphene oxide.^19^

Beyond the 24 h, the catalytic degradation of the CP triggered by the Cu nanoparticles embedded in the PCL matrix occurred. This was confirmed by evaluating the ratio between the absorbance of the characteristic UV–vis peak of the degradation product (TPC), at 321 nm, and the UV–vis absorbance peak of the chlorpyrifos, at 289 nm, at different time points, Figure S8a. The curve slope appeared after 24 h in PCL-Cu samples, while PCL displayed a straight line, which confirmed that the catalytic process did not occur.

Moreover, the high dependence on visible-UV light sources of most of the photocatalytic materials used for the removal of organic pollutants in wastewaters limits the efficiency of the catalysis in certain environmental conditions, for example, underground water or waters with suspended solids.^55^ Therefore, it was necessary to address the potential visible-UV light source dependence of these materials (Experimental Section). Figure 5b shows that the elimination efficiency of the PCL-Cu mats was similar in both light and dark conditions, indicating the independency to light and the high adaptability of the material, which could be also applied in environments with scarce or no light. The UV–vis spectra of the chlorpyrifos solution in contact with 5 mg of zerovalent Cu powder and 5 mg of PCL-2.5Cu mats, as representative samples, under dark and light conditions at different time points were reported in the Figure S9 of the Supporting Information. The scarce differences observed between the UV–vis bands can confirm that the degradation reaction evolved in light and dark conditions was similar. In agreement with the mechanism proposed in Figure 4b, in which light is not required. Moreover, this experiment evidenced that the mechanism developed in Cu-PCL mats was identical as zerovalent Cu powder, despite the chemical modifications undergone during the material fabrication.

Then, the influence of the copper content transferred to the PCL mats. The concentration (mg/L) of CP eliminated through the catalytic activity of copper vs the content in copper in samples is shown in Figure 5b. These values correspond to 7.7 mg/L for PCL-1.25Cu, 13.0 mg/L for PCL-2.5Cu, and 12.4 mg/L for PCL-5Cu. As observed, the catalytic activity is nonlinear with respect to the concentration of copper. The degradation response increased with the copper concentration up to a critical value (2.5 wt %) where the catalytic activity reached a plateau. This result was attributed to the aggregation of nanoparticles in mats at moderate concentrations of copper, decreasing the active surface exposed to the pesticide and limiting the catalytic activity.^18^ This was confirmed by the SEM micrographs of the PCL-1.25Cu, PCL-2.5Cu, and PCL-5Cu samples at higher magnifications (Figure S10).

The kinetics of the degradation reaction was evaluated by plotting ln (*C*_0_/*C*) as a function of time, and the results were reported in Figure 5c. A straight line obtained confirmed the apparent first-order kinetic law. The first order elimination rate constants were *k*_1_ = 4.19 × 10^–5^ min^–1^ (*R*^2^ = 0.98), *k*_1_ = 5.27 × 10^–5^ min^–1^ (*R*^2^ = 0.99), and *k*_1_ = 6.23 × 10^–5^ min^–1^ (*R*^2^ = 0.99), for PCL-1.25Cu, PCL-2.5Cu, and PCL-5Cu, respectively. The positive effect of copper on the degradation rate was associated with the presence of the largest number of active sites on the surface of the fibers.^52^ These values of the degradation rate were around 3 orders of magnitude below other materials with photocatalytic activity.^37,56^ However, the self-catalytic nature of these samples, which means that the catalytic activity does not require UV-light sources, is a great advantage that simplifies and streamlines the employment of the materials presented in this work.

The catalytic activity, in terms of the kinetic constant and catalytic removal (i.e., excluding the pesticide elimination by the adsorption of PCL) of the copper integrated in the fibers, in comparison with that of the free nanoparticles, was reported in Table 1. Herein, the first-order elimination rate constant, represented in Figure 5d and Figure S8b, and the percentage of catalytic removal (%) were calculated and normalized by the content of active agent (μg). For evaluating the catalytic response of the free copper nanoparticles (CuNPs), 5 mg of copper powder was packed in paper pouches and immersed in the chlorpyrifos solution (40 mg/L). After 72 h, nanoparticles have degraded all the pesticide obtaining only the degradation product (TPC). This process was repeated several times, observing the total degradation of CP in all solutions. However, after seven cycles the paper pouches broke and CuNPs leached out, hampering the calculation of the overall removal capability of free nanoparticles. Therefore, the values reported in Table 1 corresponded to the values obtained after reaching the maximum degradation point reached from 40 mg/L of pesticide (i.e., after 96 h for Cu-PCL mats and 72 h for CuNPs). The higher values of the kinetic constant (*k*_1_, min^–1^) and catalytic removal (%)/40 mg/L CP of CuNPs, with respect to the Cu-PCL mats, indicated at first sight that free nanoparticles exhibited a better catalytic response. However, the kinetic constant provided by CuNPs is only between two and four times higher than those provided by the fiber mats, whereas the amount of available CuNPs was between one and five hundreds time higher. This was attributed to the strong tendency of free metal nanoparticles to aggregate, which reduces their catalytic efficiency; however, nanoparticles anchored to the polymer exhibited a proper dispersion throughout all the electrospun microfiber, as shown in SEM images (Figure 1).^52,53^ Moreover, when normalized by the content of active agent, the performance provided by Cu-PCL mats presented higher efficiency, more specifically in samples with the lowest Cu content, ∼1.79%/μg for PCL-1.25Cu, against 1.71%/μg and 0.87%/μg, observed for PCL-2.5Cu and PCL-5Cu, respectively. These results indicated that the concentration of copper beyond a determinate quantity (2.5 wt %) can be a limiting factor for the reaction performance due to the aggregation observed in SEM images of Figure S10. Looking forward achieving the higher yields in catalytic removal of CP, PCL-2.5Cu was selected as the best sample.

**Table 1:** Content of Copper (μm), Kinetic Parameters, And Removal (%)/Content of Copper (μm) of PCL-1.25Cu, PCL-2.5Cu, PCL-5Cu, and CuNPs

Sample	Content of Cu (*μg*)	First-order elimination rate constant *k*_1_ (min^–1^)	First-order elimination rate constant *k*_1_ (min^–1^/content of Cu (*μg*)	Catalytic removal (%)/40 mg/L CP	Catalytic removal (%)/content of Cu (*μg*)
PCL-1.25Cu	12.24 ± 1.25	4.19 × 10^–5^	3.42 × 10^–6^	22.02 ± 3.21	1.79 ± 0.26
PCL-2.5CU	22.91 ± 5.97	5.27 × 10^–5^	2.22 × 10^–6^	39.36 ± 5.18	1.71 ± 0.22
PCL-5CU	42.85 ± 2.88	6.23 × 10^–5^	1.45 × 10^–6^	37.37 ± 1.09	0.87 ± 0.09
CuNPs	5770 ± 369.14	1.58 × 10^–4^	2.74 × 10^–8^	100	0.01 ± 0.00

### Reusability Study

**Figure 6:**
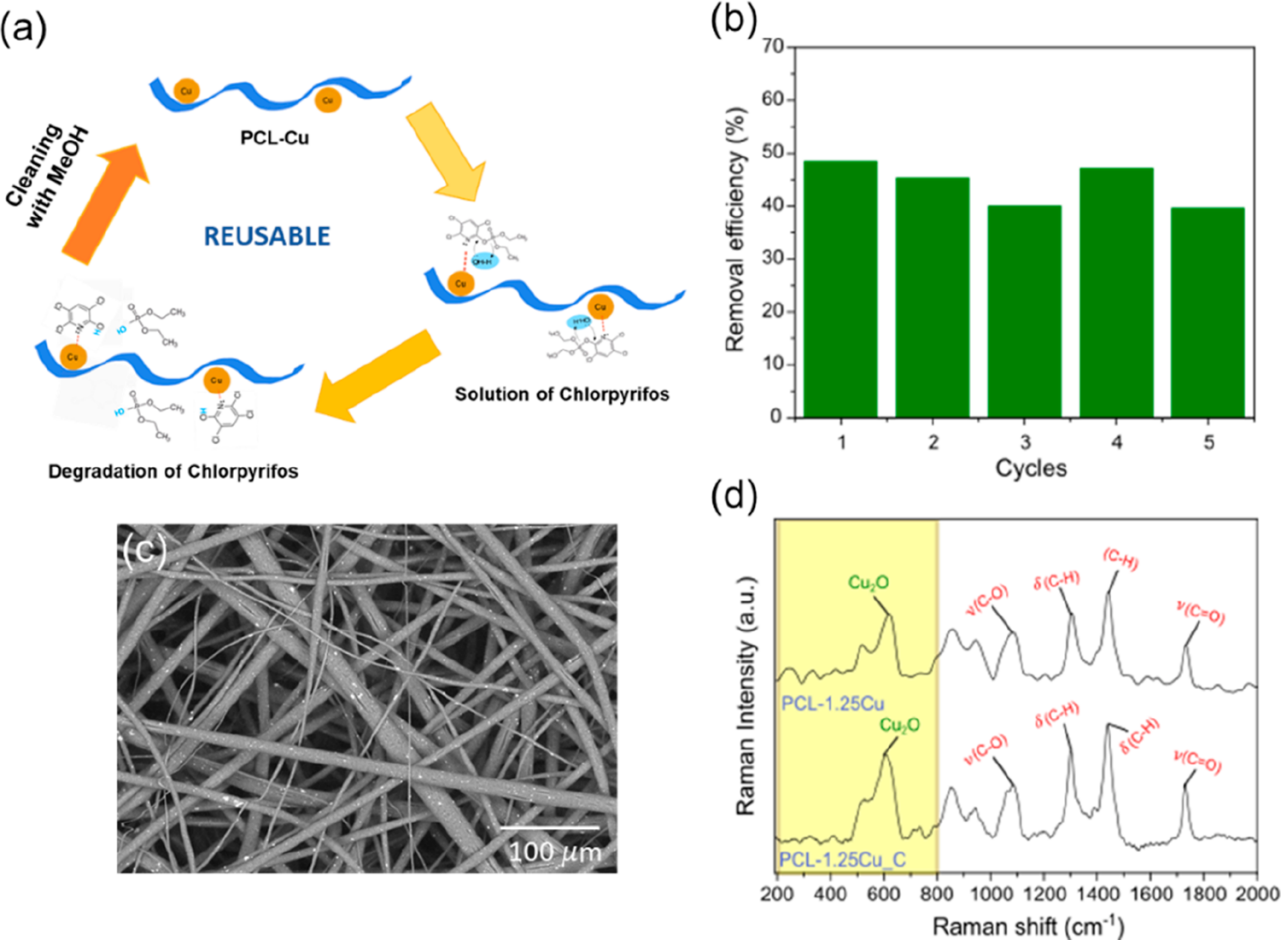
(a) Scheme of the steps for reusing the material. (b) Removal efficiency (%) of five pesticide removal cycles. Nonsignificant differences were obtained among results of each cycle according to Tukey’s test (*p* < 0.05). (c) SEM images of the PCL-2.5Cu sample after 5 cycles. (d) The Raman spectra of PCL-1.25Cu sample after 5 cycles compared to the neat PCL-1.25Cu sample.

The potential regeneration and reusability of the catalytic electrospun microfibers were evaluated by measuring the removal capability (%) for five successive cycles. As observed in the scheme represented in Figure 6a, an intermediate washing step with methanol (the sample was rinsed and shaken for 45 min) was included between each cycle. As shown previously, PCL-2.5Cu had a maximum pesticide removal of 48% after 96 h. This response remained constant during the five cycles; see Figure 6b. It is worth noticing that all the mechanisms involved in pesticide removal (adsorption and catalysis) preserved their initial performance after each pesticide degradation cycle and washing step. The complete removal of the pesticide in the wash-step was demonstrated by analyzing the infrared spectra of the PCL mats and UV–vis spectrophotometry of the solution before and after the rising; see Figure S11a, b. This reactivation method is less aggressive and faster in comparison with others found in the literature, such as exposure to high temperatures,^18,56^ centrifugation^17,37^ or acids.^57^ Interestingly, in Figure 6c, the SEM image of the samples after the five cycles, which comprise a total of 20 days, revealed that the inner morphology of the samples remained unspoilt. Besides, in Figure 6d, the Raman spectra of the PCL-1.25Cu sample taken after 5 cycles of pesticide removal (PCL-1.25Cu_C) were compared with the Raman spectra obtained in the pristine sample (PCL-1.25Cu). The lack of significant differences between the spectra indicates that the copper embedded did not undergo further oxidation toward Cu(II), with characteristic Raman peaks located at 615 cm^–1^, 540 cm^–1^, and 295 cm^–1^.^31,49^ This indicates that PCL protects also against solvent oxidation, allowing the extension of the catalytic activity for more cycles.^58^ Finally, it was proven that no release of any copper species is produced during the treatment of the polluted solutions. The absence of additional UV–vis peaks related to the surface plasmonic response of copper species in the reaction solution (Figure S12, see the Supporting Information) indicated that nanoparticles remained anchored after multiple cycles.^33,59,60^

The starting concentration of pesticide used in all cycles was constant, 40 mg/L. As said before, this concentration coincides with the maximum concentration of this pesticide found in natural waters of some cities of Bangladesh.^10^ This study indicates that almost half of this toxic pollutant can be removed in one cycle under those dramatic polluting conditions. However, the possibility of reusing this material for several cycles will allow the complete elimination of the pesticide, reaching in five cycles an accumulated chlorpyrifos removal capacity up to 100 mg/L.

## Conclusions

The PCL-Cu electrospun microfibers developed in this work have been demonstrated the successfully remove chlorpyrifos, one of the most used and dangerous pesticides worldwide. The PCL fibers were shown to retard the oxidation of copper, maintaining their excellent catalytic activity. The pesticide removal process was conducted through two mechanisms: adsorption and catalysis, driven by PCL and Cu NPs, respectively. The adsorption capability of PCL was superior to other adsorbents found in the literature. Regarding the catalytic activity, the concentration of copper was a relevant factor that upgraded the kinetic and degradation yield, up to a maximum Cu concentration of 2.5 wt % (PCL-2.5Cu sample). Interestingly, the catalytic activity was not influenced by the light irradiation, showing an independency to light conditions, allowing its use in several framework conditions. Moreover, these materials could be reused for at least five cycles, with a wash step between each cycle, maintaining the same pesticide removal efficiency and preserving the original morphology and physicochemical properties for both PCL and CuNPs. These results indicate that the PCL-Cu electrospun mat can be a potential candidate to solve the worldwide critical environmental and health problems caused by the toxic Chlorpyrifos pesticide.

## Experimental Section

### Materials

Polycaprolactone polymer (PCL, average *M*_n_ ≈ 80 000, ρ = 1.15 g cm^–3^, *T*_m_ = 61 °C) was used for the fabrication of the electrospun fibers. Chlorpyrifos (PESTANAL, analytical standard), and Copper nanopowder (Cu, average particle size 25 nm) were purchased from Sigma-Aldrich, the absence of oxidized Cu due to the storage was confirmed by Raman spectroscopy (see Supporting Information, Figure S3). Chloroform, methanol, and ethanol were purchased from Scharlab. Deionized water was obtained from a RiOs-DI 3 Water Purification device.

### Fabrication of Solid Fibers

PCL and PCL fiber mats loaded with different concentrations of CuNPs (1.25, 2.50, 5.00 wt %) were fabricated through electrospinning. Chloroform solutions of PCL (1.6 g/mL) were prepared to produce fiber mats. In addition, for the production of the PCL-Cu mats, chloroform solutions with the desired concentration of copper nanoparticles were placed into a sonication bath (Ultrasons power bath sonicator J.P. Selecta) at 50 Hz for 1 h. Then, these solutions were mixed with chloroform solutions of PCL to obtain final solutions of 6 and 1.6 g/mL PCL for all samples. The PCL-Cu solutions were shaken in a Heidolph Multi Reax Vortex mixer at 300 rpm overnight to guarantee the mixing of all components.

**Figure 7:**
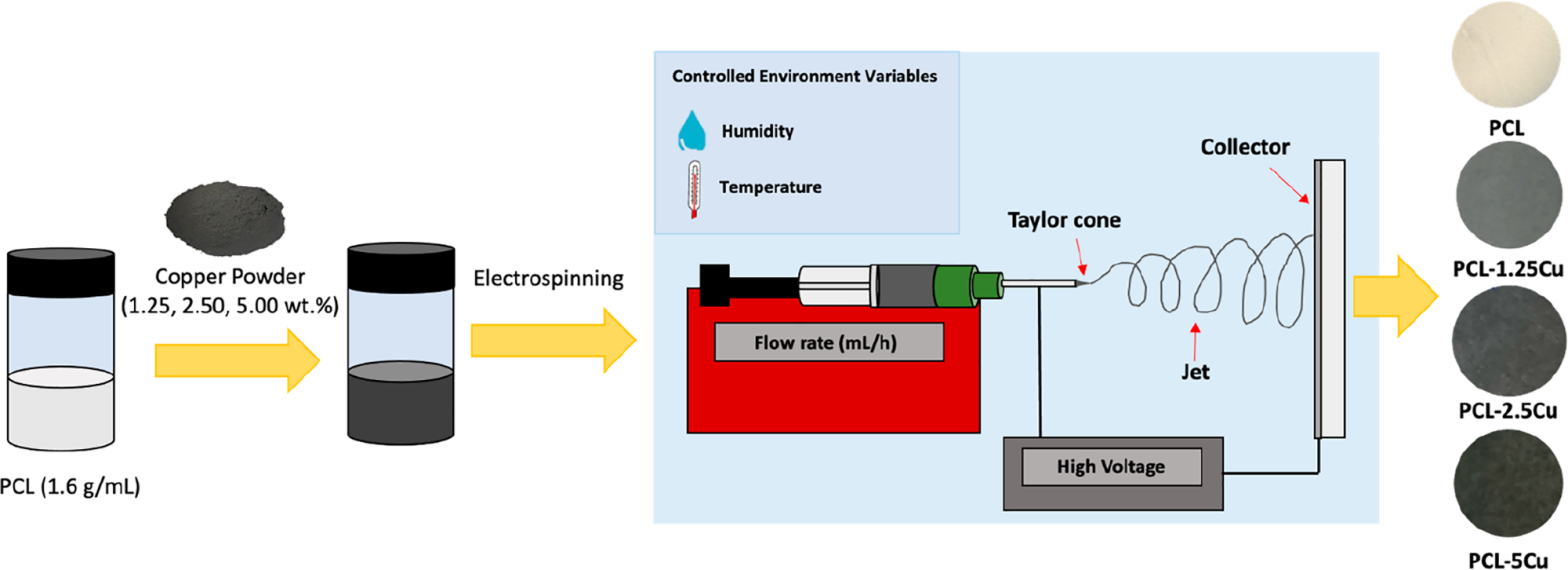
Schematic representation of the fabrication process of PCL and PCL-Cu mats obtained through the electrospinning technique.

PCL electrospun microfibers were obtained by transferring the Chloroform solutions of PCL (1.6 g/mL), or PCL (1.6 g/mL) loaded with Cu at different concentrations (1.25, 2.50, 5.00 wt % with respect to the polymer) into a syringe of 3 mL equipped with a needle 21G. The syringe was placed in a syringe pump NE-1000, New Era Pump Systems, Inc. providing a constant flow rate. The electrospun fibers were deposited on a collector that consisted of a copper target covered with aluminum foil, which was placed at a certain distance from the syringe, and a fixed voltage was applied between the needle and the collector. The process was performed under controlled environmental conditions (i.e., about 23 °C and 33% relative humidity). The scheme of the fabrication process is reported in Figure 7.

The electrospinning setup has a strong influence in the final morphology features of the fibers and include the electrical field (keV), the flow rate (mL/h), the deposition time (min), and the tip-to-collector distance (cm). These variables barely changed for each sample. The collector distance was 20 cm, while deposition time consisted of 50 min in all cases, obtaining mats with an average thickness of ∼0.3 mm, measured with a Mitutoyo 7321 Dial Thickness Gauge 0–10 mm, precision (±0.01 mm). For each sample, the electrical field and flow rate varied between 20 and 22 keV and 0.6–0.7 mL/h. All these parameters are detailed reported in the Table S2 in Supporting Information.

The selected Cu concentrations were similar to those of other electrospun microfibers loaded with nanoparticles that present catalytic activity.^61−63^ The darkening of the mats increased with the Cu concentrations, from white in the PCL mat to dark gray in the PCL-5Cu sample, see Figure 7. Besides, all mats were macroscopically homogeneous and compact, indicating the good cohesion and integrity of all elements present in the mat composite. After their preparation, mats were gently peeled off from the collector and kept under room conditions until the experiments.

Additionally, it was observed that PCL-Cu solutions stored for several days turned greenish due to copper oxidation, enabling the study of copper oxidation within these materials. Thus, these solutions were also employed for the production of PCL mats containing 5 wt % oxidized copper (PCL-5CuO_*x*_), following the method explained above, using 21 keV, 0.6 mL/h, 50 min, and 20 cm.

### Thermogravimetric Analysis

Thermal gravimetric analysis (TGA) was performed using a Mettler Toledo SDTA851 instrument. The temperature program was set in a range from 50 to 800 °C with a heating rate of 20 °C/min under a nitrogen atmosphere with a constant flow rate of 60 mL/min, followed by an isotherm at 800 °C for 15 min under an air atmosphere with a constant flow rate of 200 mL/min. At these thermal conditions, the PCL matrix degraded completely. On this basis, the quantification of copper nanoparticles was performed using the remaining residue of the thermal treatment of PCL-Cu fibers, as performed elsewhere.^64^

### Morphology Study: Scanning Electron Microscopy (SEM)

The elemental analysis of the electrospun fibers was performed through the SEM-EDX technique using a FEI ESEM Quanta 200 (Felmi-ZFE) microscope equipped with an energy-dispersive X-ray spectroscope EDAX Genesis (AMETEK Inc.) under an accelerating voltage of 15 kV and with a backscattered electron (BSE) detector to highlight the presence of copper nanoparticles. For this analysis, PCL electrospun microfibers containing copper nanoparticles were not coated. The morphological analysis was performed using a HITACHI FlexSEM 1000 scanning electron microscope. For this analysis, neat samples of PCL were coated with gold (10 nm), and the measurement was performed using an accelerating voltage of 10 kV and a secondary electron detector (SED).

### Fourier Transform Infrared (FTIR) Spectroscopy

The infrared spectra of the PCL mats were collected by using a Bruker Tensor 27 Spectrometer working in the Attenuated Total Reflectance (ATR) method with an MKII Golden-Gate accessory. Each FTIR spectrum was obtained at room temperature after 64 scans, with a resolution of 4 cm^–1^ in the range 4000–600 cm^–1^. The infrared peaks of the samples were normalized by the peak belonging to the carbonyl group of PCL located at 1720 cm^–1^.^31^

### Raman Spectroscopy

The Raman spectra were measured with a portable Raman BWTEK modular spectrometer coupled to a microscope. The spectrometer is equipped with a detector BWTEK Exemplar-Pro (resolution of 4 cm^–1^) and a laser excitation source BWTEK CleanLaze (Power Output 50–450 mW and 785 nm laser excitation wavelength). The magnification employed with the microscope was ×20. The acquisition times were 0.1–120 s, and the laser power was adjusted to ensure the innocuousness of the measurement. The equipment was calibrated with the ν(Si–Si) vibration mode, located at 520.7 cm^–1^, of a Si standard.

### XRD

X-ray diffraction (XRD) patterns were obtained with an X’Pert Pro (Malvern PANalytical) automated diffractometer using Ge(111)-monochromated CuKα radiation and an X’Celerator detector. Diffractograms were recorded between 5° and 80° (2θ) in 0.017° steps at 45 kV and 35 mA for 30 min. Samples were placed on an aluminum support adapting them to the goniometer in a θ–2θ configuration.

### XPS

XPS spectra were recorded with a Physical Electronics PHI 5700 spectrometer by using a concentric hemispherical analyzer operating in the constant pass energy mode at 29.35 eV with an analysis area of 720 μm in diameter. MgKα X-ray (*h*ν = 1253.6 eV) was used as excitation source and binding energies are referenced to adventitious C 1s peak at 284.8 eV. The residual pressure in the analysis chamber was maintained below 5 × 10^–7^ Pa during data collection. PHI ACCESS ESCA-V6.0F software package was used for acquisition and data analysis.

### Physicochemical Study of the Pesticide and Removal

The pesticide removal efficiency was evaluated for Cu powder, neat PCL electrospun nanofibers, and PCL-Cu electrospun fibers. For these experiments, 5 mg of Cu powder was introduced into wettable paper pouches to avoid the release of nanoparticles, while PCL and PCL-Cu mats were cut into 1 × 1.2 cm pieces (∼5 mg). The samples, wettable paper pouches, or pieces of the mats (1 × 1.2 cm), were immersed in 10 mL-glass containers with 5 mL of an water:ethanol:methanol (2:1:1) solution with 40 mg/L CP and shaken at a constant rape of 300 rpm at room temperature. After fixed periods of contact time (96 h), the solutions were analyzed using a UV–visible scanning spectrophotometer (UV-2600, Shimadzu), recording the samples’ absorption spectra in the range 190–400 nm. The concentrations of CP were determined employing the 289 nm peak of CP, comparing the obtained results with a calibration line (*R*^2^ = 0.99) obtained from CP stock water:ethanol:methanol (2:1:1) solutions with concentrations ranging between 10 to 45 mg/L.^22^

All experiments were performed in triplicate, and the pesticide removal efficiency (%) was calculated using eq 1:^65^
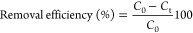
1Where *C*_0_ is the initial concentration of pesticide and *C*_f_ is the concentration at time *t*.

The removal of the pesticide by the PCL-Cu composite fibers can be carried out following multiple reaction mechanisms, like physical (e.g., adsorption), chemical (e.g., catalysis), or as a result of the action of both. Accordingly, it is necessary to address the study of the different mechanisms that participate in the overall removal of pesticide.

### Adsorption Capability of the PCL Mat

The adsorption capability (*q*) of the PCL was measured in mg of CP adsorbed by a gram of mat, and calculated by eq 2:^66^
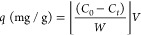
2Where *C*_0_ is the initial concentration of CP (mg/L), *C*_t_ is the remaining concentration of pesticide (mg/L) at a given time, *V* is the volume of the treated solution (L), and *W* is the weight of the sample employed as an adsorbent (g).

### Kinetic Study of Catalytic Reaction

The pseudo-first-order kinetic model was applied to determine the degradation kinetic behavior. These calculations were applied with the values obtained 24 h after the immersion of the sample in the CP solution to ensure the adsorption equilibrium prior to the catalysis response.^67^ The first-order rate constant (*k*_1_, h^–1^) was calculated by fitting the experimental data collected from a UV–vis spectrophotometer. The linear expression is given by eq 3:
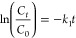
3Where *C*_0_ and *C*_*t*_ (mg/L) are the concentration of pesticide, at the initial stage and at time *t*.

Moreover, the catalytic activity of the Cu powder and PCL-Cu electrospun samples under different light conditions (photocatalysis) was assessed. For this experiment, the pesticide removal efficiency (%) was calculated by performing the reaction in vials covered with aluminum foil to simulate the dark conditions and uncovered vials to simulate visible-light conditions (exposure to a halogen lamp with visible-light irradiation ranged from 320 to 1100 nm).

### Liquid Chromatography–Mass Spectrometry (UPLC-MS-TOF) Analysis

The analysis of the formation of byproducts during the reaction was performed using Ultra Performance Liquid Chromatography (UHPLC) Acquity of Waters coupled with a Quadrupole Time-of-Flight (Q-TOF) Mass spectrometer Bruker Maxis Impact. Chromatographic separation of the aliquots was performed through UHPLC using a Kinetex C_18_ column 100A, (2.1 mm × 50 mm, 1.7 μm) of Phenomenex. The mobile phase was composed of 0.1% formic acid in water (A) and 0.1% formic acid in acetonitrile (B). The gradient program was similar to Lee et al.:^16^ 0–3 min, 5% B, 3–13 min, 5–80% B, 13–15 min, 80% B, 15–17 min, 80–5% B; and 17–20 min, 5% B. The flow rate was 0.3 mL/min, the injection volume was 5 μL, and the oven temperature was 30 °C. For profiling and identification of byproducts formed during the plasma treatment, the separated peaks were analyzed by Q-TOF providing high-resolution and mass measurement. The ESI spray voltage was set for the positive ion mode. Samples were not diluted for this analysis.

### Reusability of PCL-Cu Mats

Reusability of the fibers in the pesticide solution was demonstrated over 5 reaction cycles. After each cycle, a complete removal of the pesticides and byproducts from the fibers mats was obtained after 45 min of shaking in 5 mL of methanol. Then, the Cu-PCL fibers were immersed again in the CP solution (40 mg/L) and the removal efficiency was evaluated for the same period (4 days). Moreover, the absence of degradation signs in PCL mats after these cyclic immersions was studied with SEM microscopy.

### Statistical Analysis

Results were reported as the mean ± standard deviation. The one-way analysis of variance (ANOVA) and Tukey’s test were used to evaluate the relevance in differences among the mean values at a 0.05 level of significance.

## Supporting Information Available

SEM images of electrospun fibers at greater magnifications; XRD diffractograms and infrared spectra of samples; Raman spectra of copper powder stored; XPS CuLMM spectra; UV–vis spectra of the reaction solution for additional samples; mass spectrum of the pesticide and degradation product; UV–vis spectra of the reaction solution for the pesticide and PCL mat; ratio of UV–vis peaks and kinetic curves of the catalytic reaction of copper.; UV–vis spectra of the reaction solution for the pesticide under dark and light conditions; SEM images of electrospun fibers at greater magnifications for analyzing the distribution of CuNPs; IR and UV–vis spectra of mats before and after the washing cycle; UV–vis spectra of the reaction solution to detect the presence of CuNPs released; table with the condition and fabrication parameters of the Cu-PCL mats (PDF)
