# Supplementary material for: Encapsulation of Copper Nanoparticles in Electrospun Nanofibers for Sustainable Removal of Pesticides
Source: ACS Appl Mater Interfaces. 2023 Apr 16;15(16):20385–97. doi: 10.1021/acsami.3c00849 (PMC10141258; doi:10.1021/acsami.3c00849)
Supplement: Supplementary file 1 — am3c00849_si_001.pdf [file am3c00849_si_001.pdf]

Supporting information of the article entitled

Encapsulation of copper nanoparticles in electrospun nanofibers for sustainable removal of pesticides

Ana Isabel Quilez-Molina[1,2,], Suset Barroso-Solares[1,2,3], Violeta Hurtado-García[1,3], José Alejandro Heredia-Guerrero[4], María Luz Rodriguez-Mendez[2,5] Miguel Ángel Rodríguez-Pérez[1,2], and Javier Pinto[1,2,3,*].*

[1]Cellular Materials Laboratory (CellMat), Condensed Matter Physics, Crystallography, and Mineralogy Department, Faculty of Science, University of Valladolid, Campus Miguel Delibes, Paseo de Belén nº 7, 47011, Valladolid, Spain

[2]BioEcoUVA Research Institute on Bioeconomy, Calle Dr. Mergelina, 47011, Valladolid, Spain

[3]Archaeological and Historical Materials (AHMAT) Research Group, Condensed Matter Physics, Crystallography, and Mineralogy Department, Faculty of Science, University of Valladolid, Campus Miguel Delibes, Paseo de Belén nº 7, 47011, Valladolid, Spain

[4]Instituto de Hortofruticultura Subtropical y Mediterránea "La Mayora", Universidad de Málaga-Consejo Superior de Investigaciones Científicas (IHSM, UMA-CSIC), Bulevar Louis Pasteur 49, 29010, Málaga, Spain

[5]Group UVaSens, Escuela de Ingenierías Industriales, Universidad de Valladolid, Paseo del Cauce, 59, 47011 Valladolid, Spain

S1. Additional SEM images of electrospun fibers.

The following micrographs at higher magnifications provided a more complete vision of the morphology of the electrospun fibers. Herein, the formation of small cracks resulting from the environmental conditions of the electrospinning process is clearly observed.

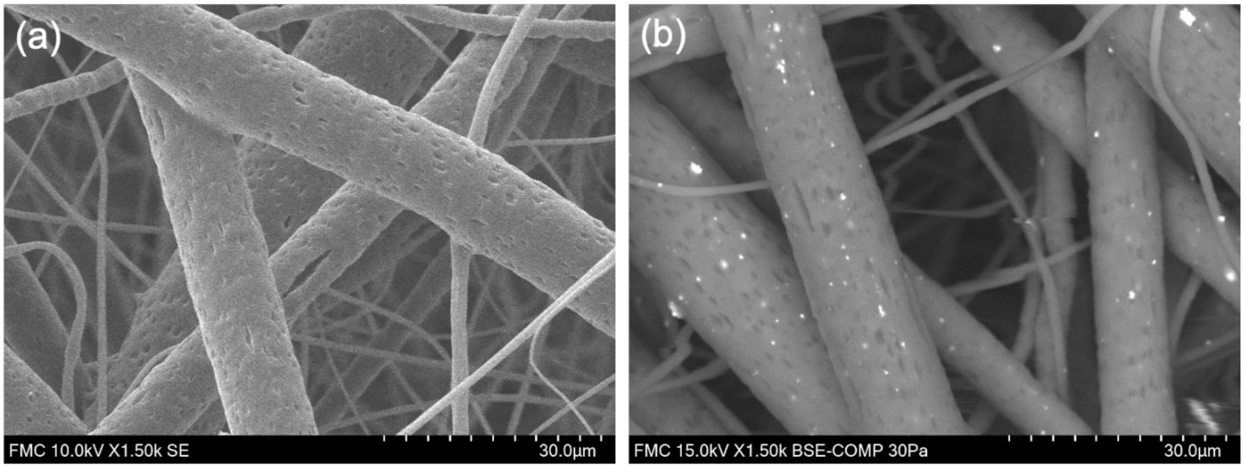

Figure S1. SEM images at high magnifications of **(a)** neat PCL and **(b)** PCL-5Cu acquired using a secondary electron detector and a backscattered electrons detector, respectively.

S2. Study of the copper oxidation through XRD and infrared spectroscopy

XRD patterns of the samples are displayed in **Figure S2(a)**. Main peaks at 21.4°, 22.0°, and 23.7°, can be ascribed to (110), (111) and (200) planes, respectively, of PCL in an orthorhombic crystal form [1]. Peaks of Cu species are masked by the PCL ones.

The study of the presence of metallic species with infrared spectroscopy is highly challenging. The infrared-assigned peaks of neat PCL and PCL-2.5Cu, and the rest of the PCL-Cu mats are presented in **Figure S2(b,c)**. The infrared spectra of all samples showed identical infrared bands typical of PCL polymer [2]. The lack of band-shifting indicated that a strong interaction between the matrix and the metallic nanoparticles was not established [3].

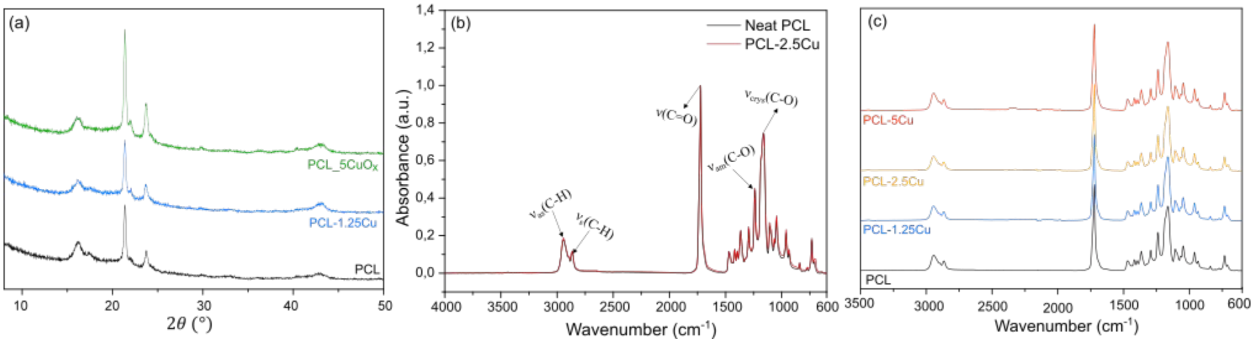

Figure S2. (a) XRD of PCL, PCL-1.25Cu, and PCL-5CuO$_x$. **(b)** Infrared spectra with assigned peaks of PCL and PCL-2.5Cu. **(c)** Infrared peaks of neat PCL, and PCL-Cu samples.

S3. Raman spectra of copper powder

The inspection of the Raman spectra confirmed the lack of oxides in the copper powder stored.

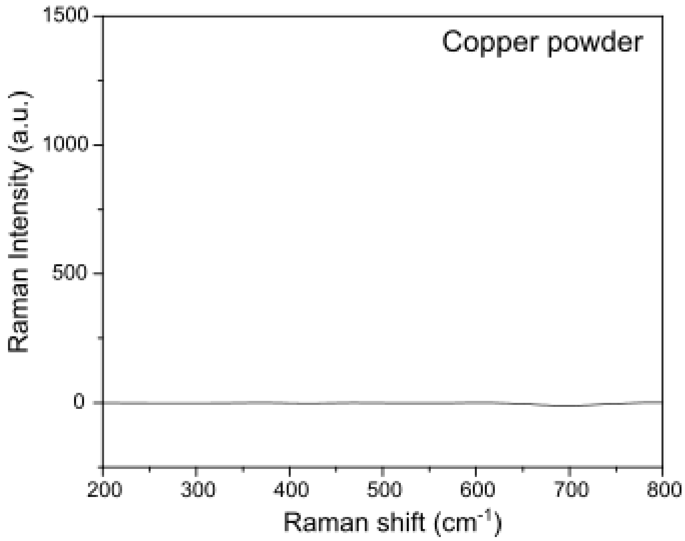

Figure S3. Raman spectra of copper powder stored. The absence of peaks corresponding to oxidizing species of copper indicates the purity of the reagent.

S4. XPS Auger signals of copper

The inspection of this section of the XPS spectra confirmed the presence of Cu(II) in PCL-5Cu_xO sample, and of Cu(I) in PCL-1.25Cu.

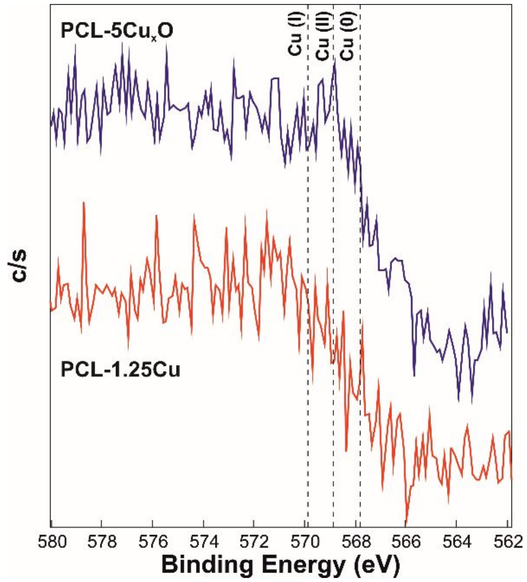

Figure S4. XPS Cu LMM spectra of PCL-1.25Cu (in red) and PCL-5Cu_xO (in blue) samples. Typical positions of Cu(0), Cu (I), and Cu (II) are included as dashed lines.

S5. The degradation mechanism of CP for PCL-Cu fibers and CuNPs studied through UV-Vis spectroscopy

The same absorbance peaks in the UV-Vis spectra of PCL-Cu fibers and free CuNPs indicated that the same chemical reaction took place. Besides, the differences in intensity over the time indicated that the content of CuNPs in PCL mats positively affect to the degradation reaction kinetics.

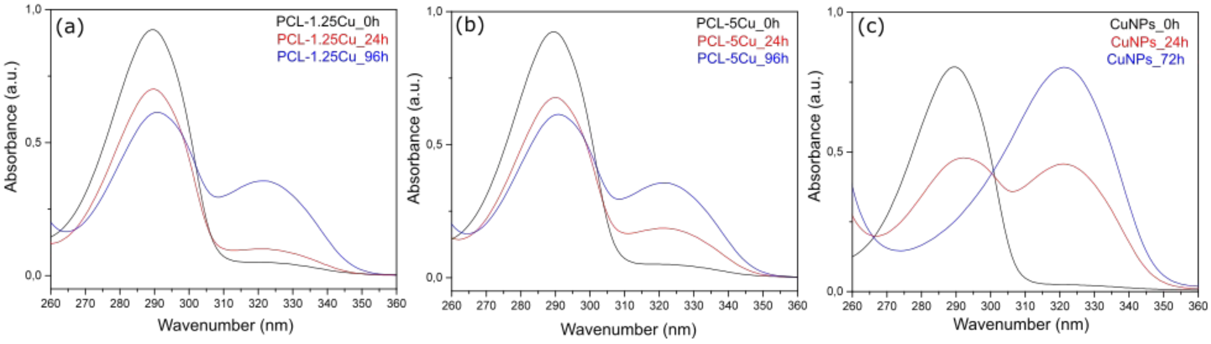

Figure S5. The UV-Vis of **(a)** PCL-1.25Cu, **(b)** PCL-5Cu, and **(c)** CuNPs after 0h, 24h, and 96h.

S6. Mass-spectrometry of the reaction solution

Mass-spectrometry spectra was used to identify the degradation products obtained in the catalytic reaction.

(a)

Analite	Meas. m/z	m/z	err [ppm]	mSigma	Ion Formula
CP	349,9339	349,9336	-0.9	13.5	C9H12Cl3NO3PS

(b)

Analite	Meas. m/z	m/z	err [ppm]	mSigma	Ion Formula
TPC	197,9279	197,9275	-2.2	11.5	C5H3Cl3NO

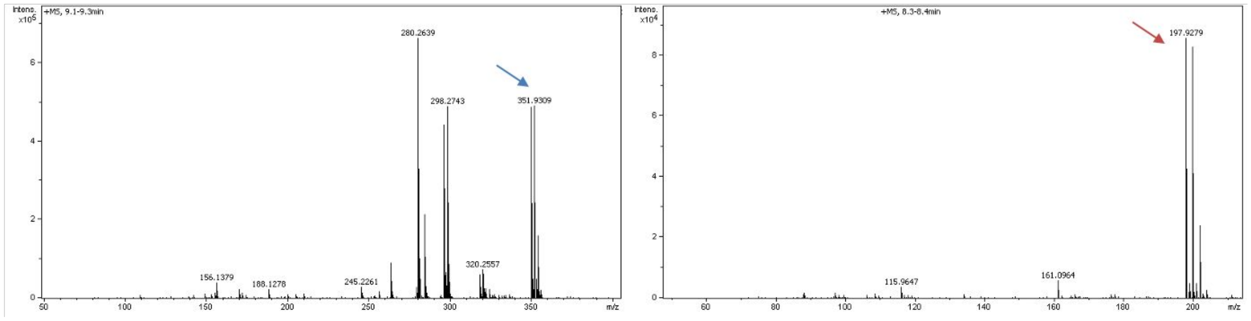

Figure S6. The mass spectrum of **(a)** Chlorpyrifos and **(b)** the degradation product TPC.

S7. UV-Vis spectra of the reaction solution over the time and after soaking the mat of neat PCL.

These spectra displayed the partial degradation of CP in solution over the time and the adsorption capability of PCL.

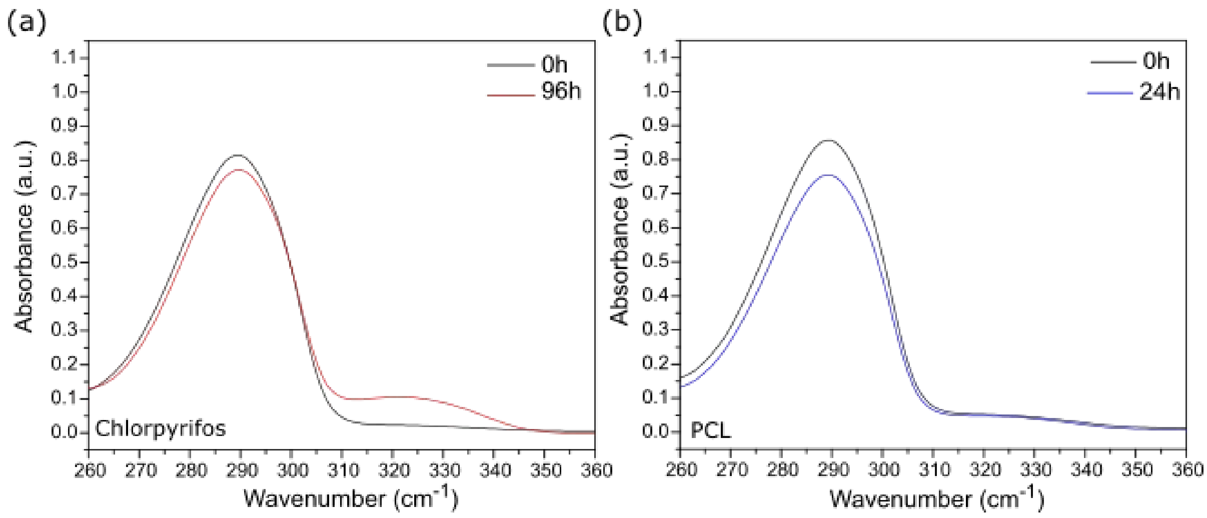

Figure S7. The UV-Vis spectra of the **(a)** Chlorpyrifos solution, at zero time-point (black), and 96 hours (red), and with **(c)** neat PCL at zero time-point (black) and after 24 hours (in blue).

S8. Study of the catalytic activity of copper for the degradation of CP

By plotting ratio between the absorbance peak associated to CP (at 289 nm) and the reaction product (at 321 nm), is possible to demonstrate that neat PCL mats did not remove the pesticide via chemical degradation. The kinetic parameter of CuNPs was obtained to evaluate the degradation efficacy PCL-Cu mats.

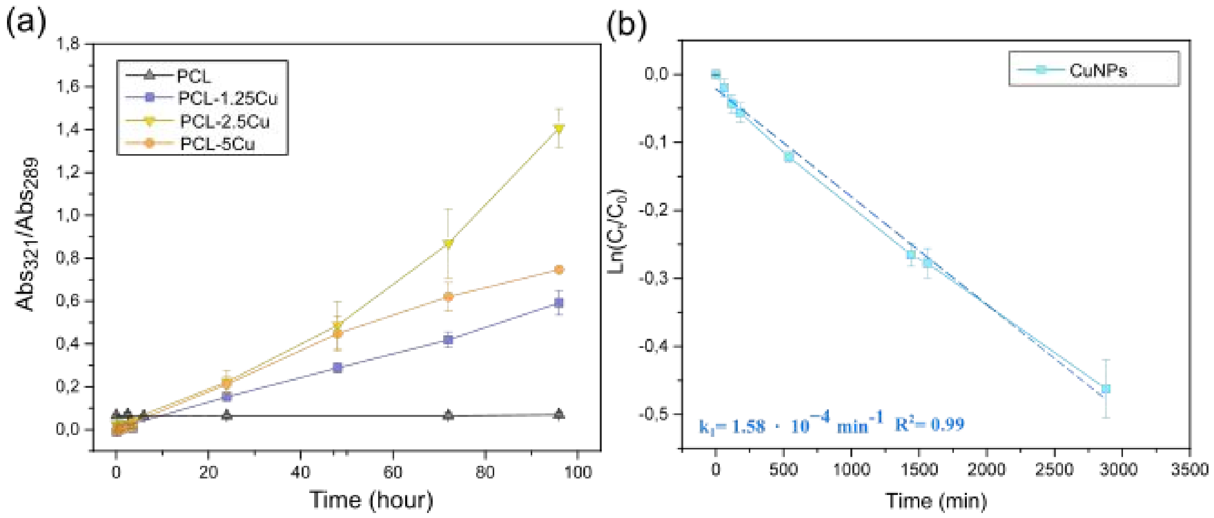

Figure S8. (a) The absorbance ratio between the UV-Vis peaks belonging to TPC (321 nm) and CP (289 nm), of PCL-Cu and PCL fibers against time (hours). **(b)** The kinetic curves of the catalytic reaction of CuNPs and the first constant rate.

S9. Study of the degradation reaction under light and dark conditions.

The UV-Vis spectra of the Chlorpyrifos solution in contact with the free copper nanoparticles and PCL-2.5Cu sample under light and dark conditions. The great similarity between the spectra at all measuring times and band overlapping indicates that the reaction was performed through the same mechanism.

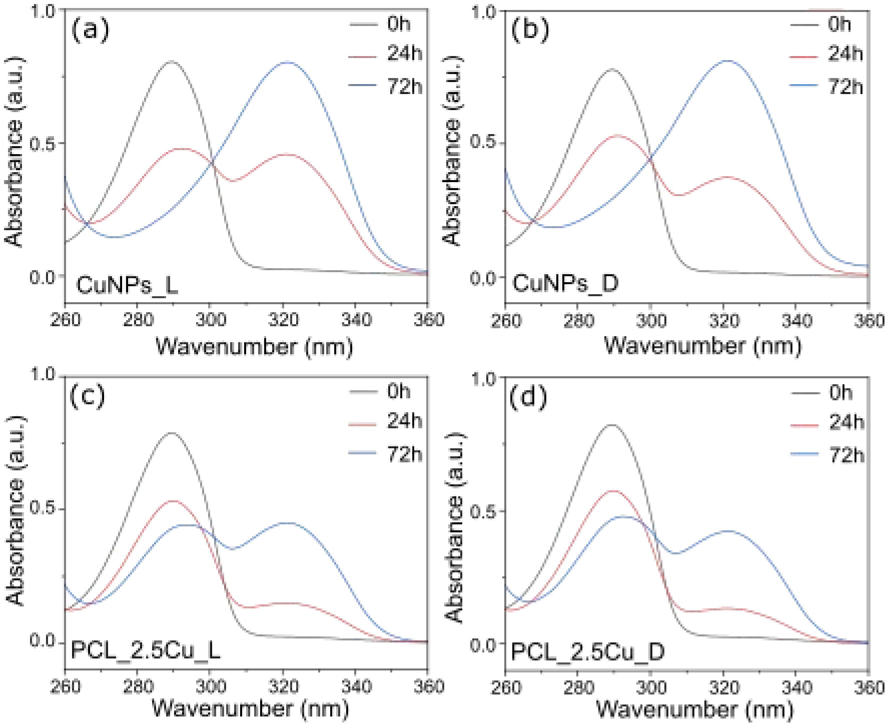

Fig. S9. UV-Vis spectra of the Chlorpyrifos solution in contact with the free copper nanoparticles (CuNPs) under **(a)** light and **(b)** dark conditions at different time points, labeled CuNPs_L and CuNPs_D, respectively. The UV-Vis spectra of the Chlorpyrifos solution in contact with PCL-2.5Cu mat at different time points under **(c)** light and **(d)** dark conditions, labeled PCL-2.5Cu_L and PCL-2.5Cu_D, respectively.

S10. Study of the particle's distribution

The micrographs at higher magnifications of the samples PCL-1.25Cu, PCL-2.5Cu, and PCL-5Cu showed that the dispersion of the nanoparticles worsened when the concentration of Cu overcame the 2.5 wt.%.

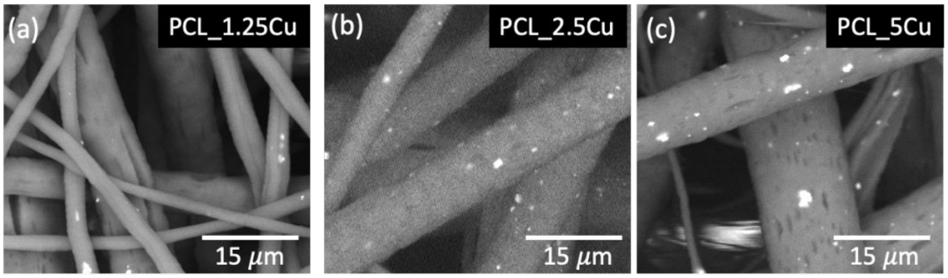

Figure S10. The SEM micrographs at high magnifications of **(a)** PCL-1.25Cu, **(b)** PCL_2.5Cu, and **(c)** PCL_5Cu.

S11. Study of the reusability of the samples

The loss of the characteristic infrared vibration peak of CP in samples after soaking in methanol indicated the complete removal of pesticide in sample. Similarly, the UV-Vis signal of CP was not present after soaking the cleaned sample in a methanol solution.

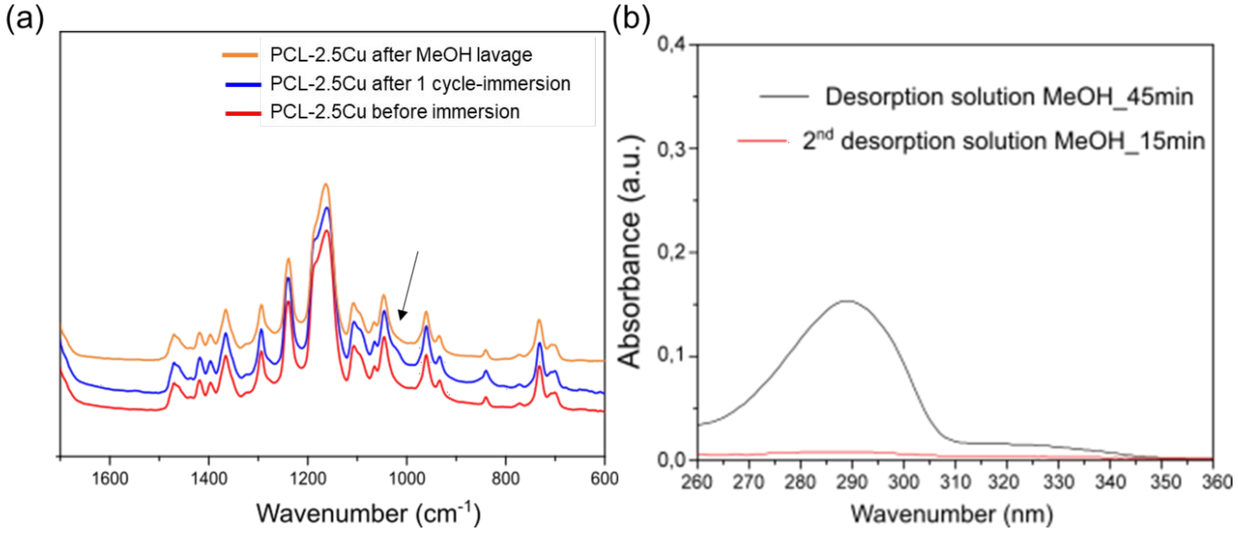

Figure S11. (a) The Infrared spectra of the PCL-2.5Cu sample before immersion (in red), after one cycle of immersion (blue), and after washing with methanol (orange). The peak corresponding to pesticide is highlighted with the black arrow. **(b)** The UV-Vis spectra of the methanol cleaning solution after 45 min of washing (black), and of the methanolic solution after the immersion of the cleaned mat for 15 min (red).

S12. Identification of CuNPs in the UV-Vis spectra

The lack of signals related to a plasmon response of copper nanoparticles indicated that CuNPs were not leached out from the PCL mats.

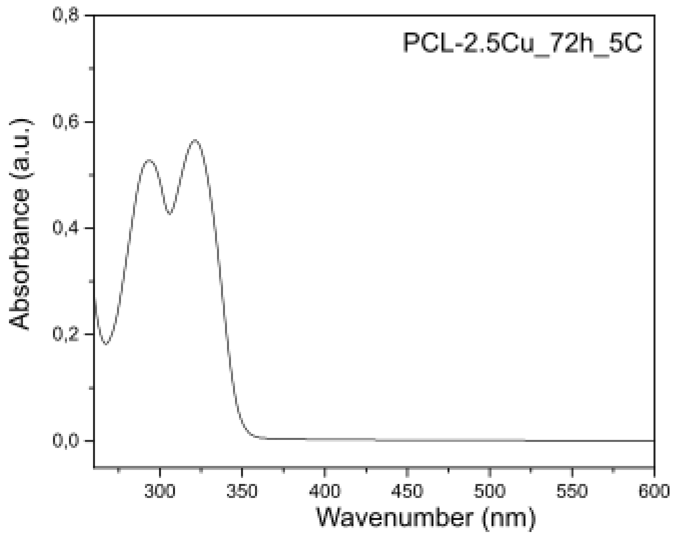

Figure S12. The UV-Vis spectra of the reaction solution of the sample PCL-2.5Cu after being immersed for 72 hours the fifth cycle. Only the peaks related to the pesticide were observed.

S13. Fabrication parameters of samples

In the preparation of the samples, the concentration of PCL in the chloroform solution was constant (1.6 g/mL), while the concentration of CuNPs added varied from 0 to 5 wt.% with respect to the polymer content. The compositional variability of samples resulted into different fabrication parameters.

Table S2. Composition and fabrication parameters of the mats studied in this work.

Sample labelling	PCL concentration (g/mL) in solution	Cu concentration (wt.%) with respect to PCL in solution	Fabrication parameters
**PCL**	1.6	0	20 keV, 0.6 mL/h, 50 min, 20 cm
**PCL-1.25Cu**	1.6	1.25	22 keV, 0.7 mL/h, 50 min, 20 cm
**PCL-2.5Cu**	1.6	2.5	20 keV, 0.6 mL/h, 50 min, 20 cm
**PCL-5Cu**	1.6	5	20 keV, 0.6 mL/h, 50 min, 20 cm
